# Estrogen negatively regulates the renal epithelial sodium channel (ENaC) by promoting Derlin-1 expression and AMPK activation

**DOI:** 10.1038/s12276-019-0253-z

**Published:** 2019-05-21

**Authors:** Xue Zhang, Yamei Ge, Ashfaq-Ahmad-Shah Bukhari, Qian Zhu, Yachen Shen, Min Li, Hui Sun, Dongming Su, Xiubin Liang

**Affiliations:** 1 Department of Pathophysiology, Nanjing Medical University, Nanjing, Jiangsu Province China; 2 Center of Pathology and Clinical Laboratory, Sir Run Run Hospital, Department of Pathology, Nanjing Medical University, Nanjing, Jiangsu Province China; 3 State Key Laboratory of Reproductive Medicine, Nanjing Medical University, Nanjing, Jiangsu Province China

**Keywords:** Hypertension, Experimental models of disease

## Abstract

The main functions of the epithelial sodium channel (ENaC) in the kidney distal nephron are mediation of sodium and water balance and stabilization of blood pressure. Estrogen has important effects on sodium and water balance and on premenopausal blood pressure, but its role in the regulation of ENaC function is not fully understood. Female Sprague–Dawley rats were treated with 17β-estradiol for 6 weeks following bilateral ovariectomy. Plasma estrogen, aldosterone, creatinine, and electrolytes were analyzed, and α-ENaC and derlin-1 protein expression in the kidney was determined by immunohistochemistry and western blotting. The expression levels of α-ENaC, derlin-1, AMPK, and related molecules were also examined by western blotting and real-time PCR in cultured mouse renal collecting duct (mpkCCDc14) epithelial cells following estrogen treatment. Immunofluorescence and coimmunoprecipitation were performed to detect α-ENaC binding with derlin-1 and α-ENaC ubiquitination. The results demonstrated that the loss of estrogen elevated systolic blood pressure in ovariectomized (OVX) rats. OVX rat kidneys showed increased α-ENaC expression but decreased derlin-1 expression. In contrast, estrogen treatment decreased α-ENaC expression but increased derlin-1 expression in mpkCCDc14 cells. Moreover, estrogen induced α-ENaC ubiquitination by promoting the interaction of α-ENaC with derlin-1 and evoked phosphorylation of AMPK in mpkCCDc14 cells. Our study indicates that estrogen reduces ENaC expression and blood pressure in OVX rats through derlin-1 upregulation and AMPK activation.

## Blood pressure: Estrogen maintains the balance

Estrogen treatment could prove valuable in tackling high blood pressure (hypertension) in postmenopausal women. Long-term healthy blood pressure is linked to the correct regulation of sodium and water levels in the kidneys. The renal epithelial sodium channel (ENaC) is a cellular membrane channel responsible for mediating sodium reabsorption and fluid balance. Liang and co-workers at Nanjing Medical University in Nanjing, China, conducted experiments on postmenopausal rat models, and found that loss of estrogen elevates systolic blood pressure (the pressure during heart muscle contraction), and that the rats had high levels of ENaC expression. Further investigations showed that estrogen treatment restored blood pressure to normal levels by promoting two key proteins involved in cellular membrane health and energy metabolism. This in turn reinstated normal levels of ENaC breakdown in the kidneys, limiting hypertension.

## Introduction

Hypertension is a major risk factor for cardiovascular disease and is also the main factor for morbidity and mortality in postmenopausal women^1^. After menopause, the incidence of hypertension significantly increases to a rate that equals or surpasses that in males, suggesting that ovarian hormones protect premenopausal blood pressure^2,3^. Among the multiple mechanisms that have been proposed to explain hypertension in menopausal women, sex hormones have been shown to have important effects on the regulation of sodium and body fluids and on the increase in blood pressure in postmenopausal women associated with sodium and water retention^4^.

The body maintains long-term stability of blood pressure by regulating water and sodium levels in the kidneys^5^. Sodium reabsorption in the distal nephron is mainly mediated by the renal epithelial sodium channel (ENaC), a sodium-selective ion channel comprising three homologous subunits (α, β, and γ), each composed of two membrane-spanning domains^6^. ENaC is widely expressed in the epithelial cells of several tissues, including the kidneys, airways, salivary ducts, sweat ducts, colon, and taste cells, and is also the limiting factor in the reabsorption of sodium across these epithelia. The kidneys adjust ENaC activity in conjunction with Na^+^-K^+^ pump activity in the distal tubule epithelial cells and cortical collecting duct (CCD)^7^.

ENaC activity is regulated by several cellular mechanisms, including channel synthesis, intercellular trafficking, and channel endocytosis^8^. The physiological functions of ENaC are modulated by the actions of several hormones (e.g., aldosterone, insulin, and vasopressin) and by nonhormonal mechanisms through intercellular proteases. ENaC expression and activity depend on ENaC synthesis and degradation, and the lysosome-mediated degradation pathways of ENaC have been well investigated^9^. ENaC internalization from the plasma membrane and ENaC degradation are controlled by Nedd4-2, an E3 ubiquitin ligase, which physically interacts with PY motifs on the C-termini of ENaC^10^. Nedd4-2 is therefore a critical target for many regulatory proteins of ENaC, including serum- and glucocorticoid-induced kinase (SGK1) and glucocorticoid-induced leucine zipper protein (GILZ1)^11,12^. SGK1 blocks Nedd4-2/ENaC binding, and GILZ1 decreases the affinity of ENaC for Nedd4-2, thereby reducing ENaC ubiquitination and increasing ENaC stability and expression at the plasma membrane^13,14^. Conversely, AMP-activated kinase (AMPK), a metabolic sensor, serves as a negative regulator of ENaC by enhancing ENaC binding with Nedd4-2 and enhancing ENaC retrieval from the plasma membrane^15,16^.

The ENaC ubiquitination and degradation mechanism involves proteasome-mediated degradation of ENaC subunits^17^. We previously showed that derlin-1, an E3 ubiquitin ligase modulator, suppressed the expression of ENaC at the protein level and promoted ubiquitination and degradation of ENaC^18^. The aims of our present study were to determine whether estrogen is involved in ENaC regulation in mammalian kidney cells and to conduct an in-depth investigation of the molecular mechanisms of ENaC regulation by estrogen.

## Materials and Methods

### Reagents, plasmid constructs and antibodies

MG132 was obtained from Enzo Life Science (NY), and 17β-estradiol (E_2_) was purchased from Sigma-Aldrich (St. Louis, MO). An antibody against α-ENaC was obtained from GenScript, as previously described^19,20^. Antibodies against derlin-1 and HA-tag were purchased from Sigma-Aldrich. Antibodies specific for ubiquitin, p-AMPK, and AMPK were purchased from Cell Signaling Technology (Danvers, UK). β-Actin, α-tubulin, and GAPDH antibodies were purchased from Proteintech (Chicago, IL). Other antibodies, including normal rabbit IgG for immunoprecipitation and secondary mouse or rabbit antibodies for western blotting, were purchased from Thermo Fisher (Waltham, UK). shRNA-derlin-1 and shRNA-scramble constructs were designed and cloned into the pcDNA3.1 vector. HA-tagged WT-AMPK-α1 or DN-AMPK, a dominant-negative (DN) AMPK-α1-K45R mutant, were gifts from Dr. Kenneth Hallows (University of Southern California). Wild-type ubiquitin was amplified by PCR and cloned into pcDNA3.1. All plasmid constructs were confirmed by DNA sequencing.

### Animal models and estrogen replacement

Female Sprague–Dawley (SD) rats weighing 280–300 g were purchased from the Laboratory Animal Center of Nanjing Medical University at 9 to 10 weeks of age. The rats were housed under a 12 h light/dark cycle at a temperature of 24 °C and a relative humidity of 56 ± 10%. The rats were randomly assigned to three groups (n = 4 per group): a group subjected to sham surgery (sham group), a group subjected to ovariectomy (OVX; OVX group), and a group subjected to ovariectomy with daily abdominal subcutaneous injections of 200 µg of E_2_ (OVX+E_2_ group). The OVX surgery of rats was conducted as previously described^21^. Systolic blood pressure (SBP) and body weight were measured at 1 week before the experiment and at 4 and 6 weeks after surgery. Systolic blood pressure was measured with a standard tail cuff method (BP-2000, Visitech Systems). All rats were sacrificed at the end of the experiment, and blood and kidney samples were obtained.

Plasma E_2_, aldosterone (ALD), serum creatinine (Cr), Na^+^, K^+^, and Cl^−^ levels were measured with an automated biochemical analyzer (7600-DDP-ISE; Hitachi Software Engineering, Yokohama, Japan) in the core lab of the Sir Run Run Hospital of Nanjing Medical University. One part of the kidneys was fixed in 10% phosphate-buffered formalin and then embedded in paraffin for histological studies. The remaining kidney tissues were snap-frozen in liquid nitrogen and stored at −80 °C for protein extraction. All animal experiments were carried out in accordance with the Guide for the Care and Use of Laboratory Animals and were approved by the Institutional Animal Care and Use Committee of Nanjing Medical University.

### Cell culture and cell transfection

Mouse cortical collecting duct (mpkCCDc14) cells (provided by Alain Vandewalle and Marcelle Bens, INSERM, Paris, France) were cultured in flasks (passage 30-40) in defined media consisting of equal volumes of DMEM and F12 containing 2% FBS, hormones and other nutrients, as previously described^18,19,22^, at 37 °C and 5% CO_2_. For cell transfection, the constructed vectors were transfected into mpkCCDc14 cells using X-tremeGENE HP reagent (Roche). At 6 h after transfection, the medium was complemented with blood serum, and the cells were maintained in an incubator at 37 °C and 5% CO_2_. After gene transfection and cell starvation treatment, the cells were treated with E_2_, and gene expression or knockdown was examined by western blotting.

### Western blotting and coimmunoprecipitation assays

Lysates harvested from mpkCCDc14 cells were resolved by 10% SDS-PAGE on gels that contained 50 mM Tris-HCl (pH 6.8), 10% ammonium persulfate, 30% Acr-Bis, 10% SDS, and 1% TEMED. Unbound sites were blocked for 1.5 h at room temperature with 5% nonfat milk in Tris-buffered saline (TBS) containing 0.1% Tween-20. The blotted membranes were probed with the indicated primary antibodies at appropriate dilutions and then incubated with horseradish peroxidase (HRP)-conjugated secondary antibodies. The immuno-recognition signals were detected using enhanced chemiluminescence (ECL) and were acquired using an Image Quant ECL system (PerkinElmer Life Sciences, Wellesley, MA). The western blotting data were quantified with Image Lab software.

For immunoprecipitation, lysates were collected from mpkCCDc14 cells that had been subjected to transient transfection or drug stimulation and lysed with NP40 containing 10 mM Tris-HCl (pH 7.4) and 10 mM NaCl. The protein concentrations of the lysates were determined by protein assays (BCA), and 0.5 mg of protein was incubated with specific antibodies overnight at 4 °C before incubation with 100 μl of protein A beads for 2–6 h. The immunocomplexes were washed with NP40, degenerated with 2 × SDS loading buffer, and subjected to immunoblotting.

### Immunofluorescence staining

The mpkCCDc14 cells were starved for 12 h before the experiment. After drug stimulation, the adherent cells were fixed in 5% paraformaldehyde and permeabilized with 5% Triton X-100 in phosphate-buffered saline (PBS) at room temperature. The cells were washed three times with PBS and then blocked with 1% bovine serum albumin for 30 min. The cells were incubated with specific antibodies at 4 °C overnight and were then incubated with fluorescent secondary antibodies for 1 h at room temperature. After washing with PBS and adding 50% glycerol, the cells were examined by fluorescence microscopy.

### Short-circuit current recordings

Polarized mpkCCDc14 cells were cultured onto permeable filter supports and mounted in modified Ussing chambers (Corning Costar). The cultures were then continuously short-circuited with an automatic voltage clamp (Department of Bioengineering, University of Iowa, Iowa City, IA), as previously described^23,24^. Amiloride (10 μM) was added to the apical bath to analyze ENaC-mediated transepithelial currents. Transepithelial resistance was calculated using Ohm’s law from the current response to a periodic 2.5 mV bipolar pulse. The bathing Ringer solution consisted of 120 mM NaCl, 25 mM NaHCO_3_, 3.3 mM KH_2_PO_4_, 0.8 mM K_2_HPO_4_, 1.2 mM MgCl_2_, 1.2 mM CaCl_2_, and 10 mM d-glucose. The chambers were maintained at 37 °C and gassed continuously with a mixture of 95% O_2_ and 5% CO_2_, which fixed the pH at 7.4.

### RNA extraction and real-time PCR

Total RNA was extracted from mpkCCDc14 cells in TRIzol reagent (Invitrogen) following the manufacturer’s instructions. The total RNA was reverse transcribed using a commercial kit (Takara). A 10 μl mixture containing 1000 ng of RNA and a set of gene-specific primers were subjected to real-time PCR using an ABI StepOnePlus System (Bio-Rad). The sequences of the primers used in the qPCR were as follows: α-ENaC, forward, 5′-TGTGTCCAGCTACAAACCAATG-3′ and reverse, 5′-CATCATGCCCACTTCGTAACA-3′; derlin-1, forward, 5′-CATCACGCGCTACTGGTTTG-3′ and reverse, 5′-GAACGGCCTCCATATCTGGAA-3′; and arpppo, forward, 5′-GAAACTGCTGCCTCACATCCG-3′ and reverse, 5′-GCTGGCACAGTGACCTCACACG-3′.

### Statistical analysis

Statistical analyses were performed using SPSS 13.0 software. All data are presented as the mean ± SE. Differences in the various parameters among the groups were evaluated by ANOVA with post hoc comparisons tests. Statistical significance was defined as *P* *<* 0.05.

## Results

### The loss of estrogen elevates systolic blood pressure in OVX rats

**Fig. 1:**
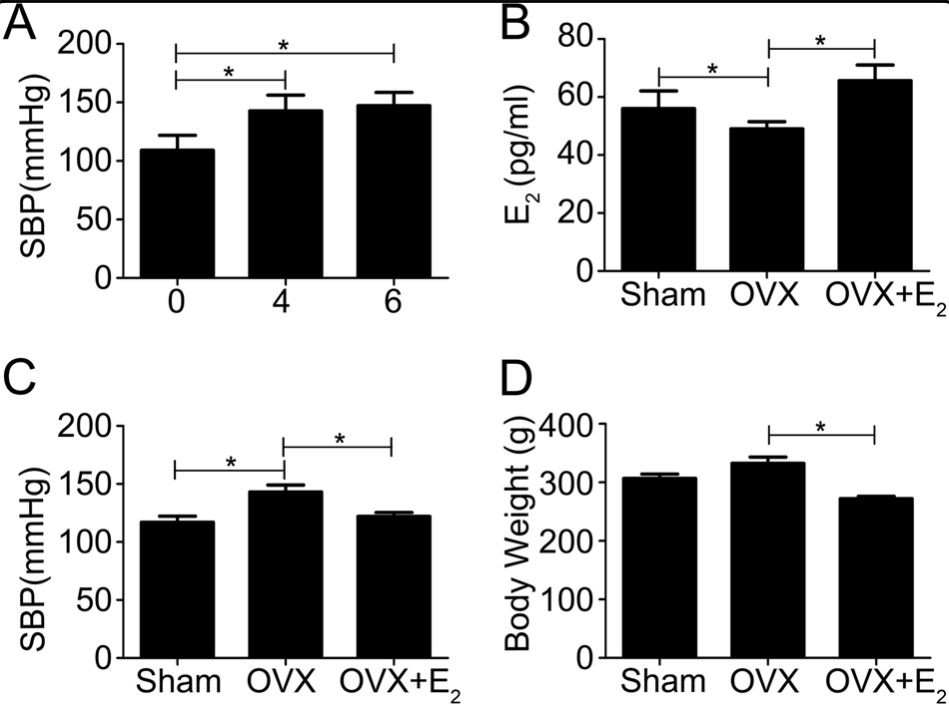
Estrogen deficiency elevates systolic blood pressure in OVX rats. **a** OVX rats demonstrated significantly increased SBP 4 weeks and 6 weeks after OVX compared to sham rats. **b** Plasma estrogen levels dramatically decreased after the OVX procedure, but 17β-estradiol (E_2_) replacement restored plasma estrogen levels in OVX rats. **c** E_2_ treatment significantly attenuated the OVX-induced increases in SBP levels to near control levels. **d** E_2_ replacement markedly decreased body weight in OVX rats. The bars are the means ± SE values from three independent experiments. *P *<* 0.05 (*n* = 5 per group)

The influence of estrogen on SBP was determined by subjecting SD rats to bilateral ovariectomy (OVX). SBP was significantly increased in OVX rats at 4 weeks and 6 weeks after OVX compared to sham rats (Fig. 1a).

OVX rats showed dramatically decreased plasma estrogen levels after the OVX procedure, but subcutaneous injection of 17β-estradiol (E_2_) restored the plasma estrogen levels of OVX rats to control levels (Fig. 1b). Estrogen treatment also significantly decreased the OVX-induced increase in SBP to levels comparable to those observed in nonoperated sham female rats (Fig. 1c). Hormone replacement with E_2_ also markedly decreased the body weight of OVX rats (Fig. 1d). These findings confirmed the protective effects of estrogen on body fluid levels as well as blood pressure in rats.

Table 1 shows that plasma ALD levels were significantly higher in the OVX group than in the control group and that E_2_ treatment in the OVX+E_2_ group effectively prevented this increase in ALD levels. No statistically significant differences were noted for the values of serum Cr, K^+^, or Cl^−^ among the control, OVX, and OVX+E_2_ groups. In contrast, a significant elevation in serum Na^+^ was observed in OVX rats that was attenuated by E_2_ addition in OVX+E_2_ rats. These data indicated that E_2_ might exert its protective effect on SBP by maintaining the balance of Na^+^ reabsorption, body fluid levels, and blood pressure through the regulation of renal sodium transporters.

**Table 1:** Renal functional and electrolytic parameters in rats

	Sham	OVX	OVX+E_2_
ALD (pg/mL)	227.3 ± 19.3	289.8 ± 23.4*	234.1 ± 24.2^#^
Cr (μmol/L)	21.7 ± 2.4	23.3 ± 3.6	22.1 ± 2.9
Na^+^ (mmol/L)	138.6 ± 2.2	147.3 ± 1.3*	142.5 ± 1.2^#^
K^+^ (mmol/L)	5.3 ± 0.72	5.1 ± 0.39	5.8 ± 0.28
Cl^−^ (mmol/L)	102.5 ± 3.7	101.8 ± 3.2	101.7 ± 4.1

*ALD* aldosterone, *Cr* creatinine

*P < 0.05 VS Sham; ^#^*P* < 0.05 VS OVX

### Ovariectomy increases the expression of α-ENaC in the kidneys of rats

**Fig. 2:**
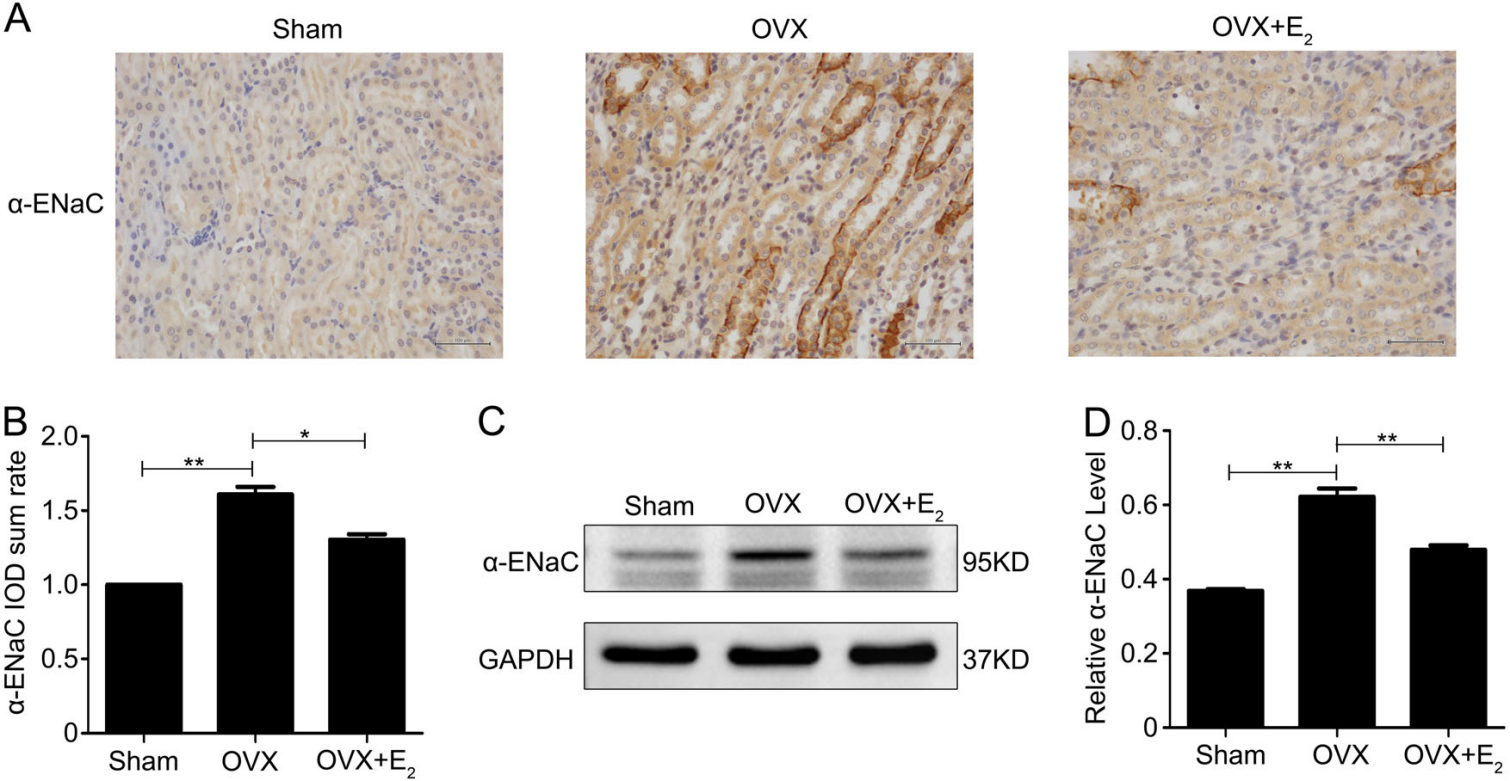
Estrogen deficiency increases the expression of α-ENaC in the kidneys of rats. **a** The expression of α-ENaC protein in kidney tissues of rats subjected to different treatments was detected by immunohistochemical staining. **b** Quantification of the positive staining for the α-ENaC protein in (**a**). **c** Western blot analysis was performed to confirm the expression of α-ENaC in kidney lysates from the different treatment groups. **d** The intensity of the α-ENaC protein bands was analyzed by densitometry as in **c** and normalized to the GAPDH level. The bars are the means ± SE values from three independent experiments. *P *<* 0.05 and **P *<* 0.01

ENaC plays a key role in maintaining sodium balance in the kidney. Immunohistochemical staining for the expression of the α-ENaC protein in rat kidney tissues indicated that the positive signals for α-ENaC protein were predominantly localized in the renal tubular epithelium and collecting ducts in control rats, and this staining was increased in OVX rat kidneys (Fig. 2a). Treatment with E_2_ in OVX rats attenuated the increase in α-ENaC protein expression (Fig. 2b). α-ENaC protein expression, as determined by western blotting of kidney protein lysates, was greater in the kidneys of OVX rats than in those of control rats but was similar in the kidneys of OVX+E_2_ rats and controls (Fig. 2c). The quantified western blotting data normalized to GAPDH expression from all experiments are shown in Fig. 2d. These data indicated that E_2_ treatment may affect sodium reabsorption by limiting renal ENaC expression.

### Estrogen suppresses the expression of ENaC in mpkCCDc14 cells

**Fig. 3:**
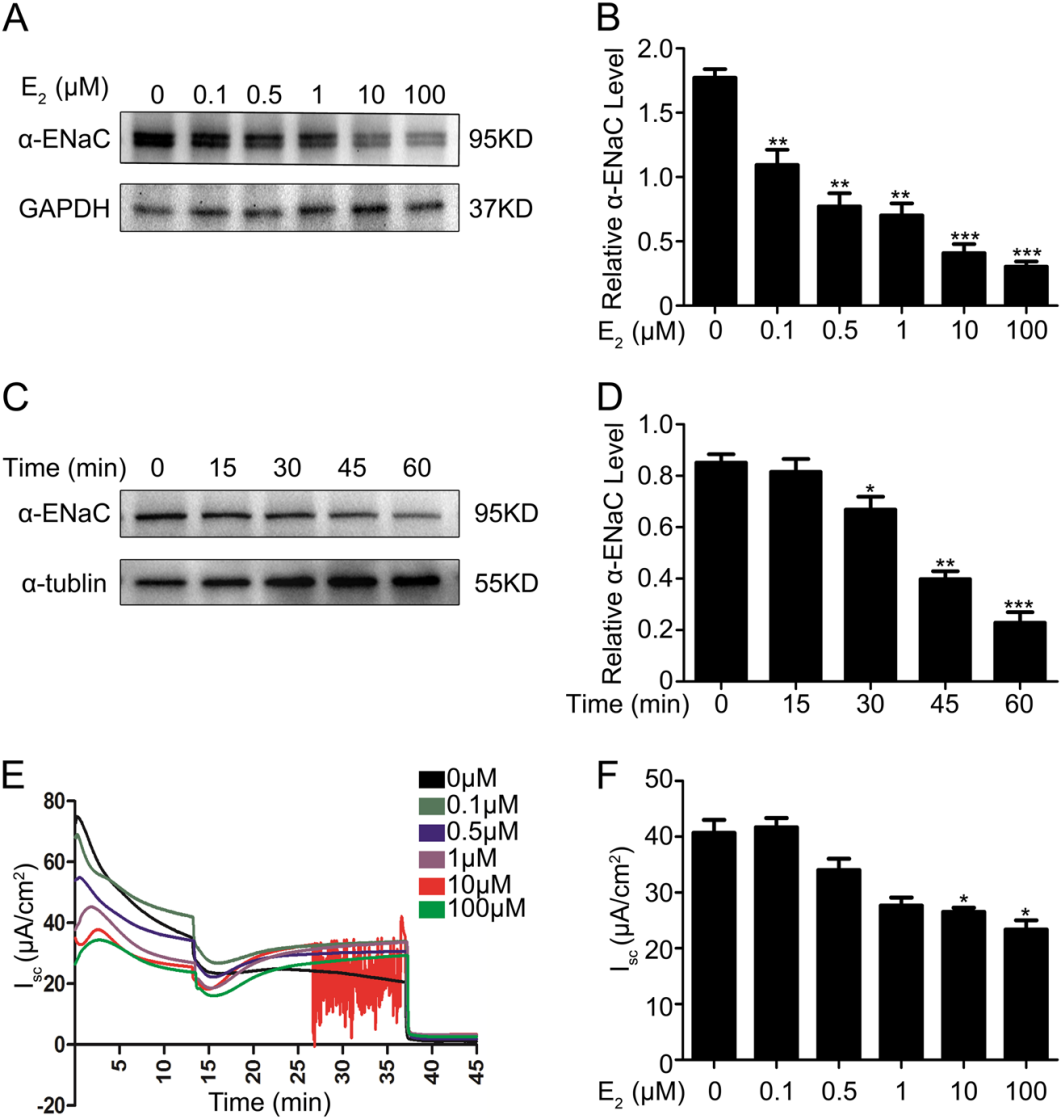
Estrogen suppresses the expression of ENaC in mpkCCDc14 cells. **a** Endogenous α-EnaC expression was dose-dependently decreased by E_2_ treatment in mpkCCDc14 cells, as determined by western blot analysis. **b** Quantitation of the western blot data for the EnaC protein from **a**. The bars are the means ± SE values from three independent experiments. *P *<* 0.05, **P *<* 0.01, and ***P *<* 0.001 compared to the Con group. **c** E_2_ suppressed the protein expression of α-ENaC in mpkCCDc14 cells during a time course, as detected by western blot analysis. **d** The mean time-course data for α-ENaC expression in C normalized to α-tubulin expression. The bars are the means ± SE values from three independent experiments. *P *<* 0.05, **P *<* 0.01, and ***P *<* 0.001 compared to the Con group. **e** Typical traces of short-circuit current (Isc, μA/cm^2^) across mpkCCDc14 cell epithelia under E_2_ treatment at a series of concentrations. The *arrows* indicate the time of addition of 10 μM amiloride to the apical bath. **f** Amiloride-sensitive Isc from all experiments of the type shown in (**e**); the data are from four epithelia studied under each condition. *P *<* 0.05 compared to the Con group

Direct evidence for a relationship between E_2_ and ENaC was obtained by examining the modulatory effect of E_2_ on α-ENaC expression in cultured mpkCCDc14 cells. Treatment of these cells with 0.1, 0.5, 1, 10, or 100 µM E_2_ resulted in a dose-dependent decrease in endogenous α-ENaC expression (Fig. 3a). The quantified immunoblot data normalized to GAPDH expression from all experiments are shown in Fig. 3b. Treatment with 100 µM E_2_ resulted in a time-dependent suppression of α-ENaC expression in mpkCCDc14 cells, as determined by western blotting (Fig. 3c), and these data are summarized in Fig. 3d.

The functional impact of E_2_ on transepithelial Na^+^ absorption mediated by ENaC was determined by measuring the amiloride-sensitive short-circuit current (Isc) across mpkCCDc14 cells under control conditions or following treatment with different doses of E_2_ for 24 h. Representative Isc traces are shown in Fig. 3e, and the mean data are shown in Fig. 3f. E_2_ treatment induced a dose-dependent decrease in the amiloride-sensitive Isc across mpkCCDc14 cells, which is consistent with the biochemical findings shown in Fig. 3a and confirms a physiological role for estrogen in the negative regulation of sodium transport. Real-time PCR also revealed enhanced α-ENaC mRNA expression in mpkCCDc14 cells treated with E_2_. Supplementary Fig. 1a and b show significant dose- and time-dependent decreases in the mRNA levels of α-ENaC following treatment of mpkCCDc14 cells with E_2_. These findings indicated a regulation of α-ENaC expression by E_2_ at both the mRNA and protein levels.

### Ovariectomy decreases the expression of derlin-1 in rat kidneys

**Fig. 4:**
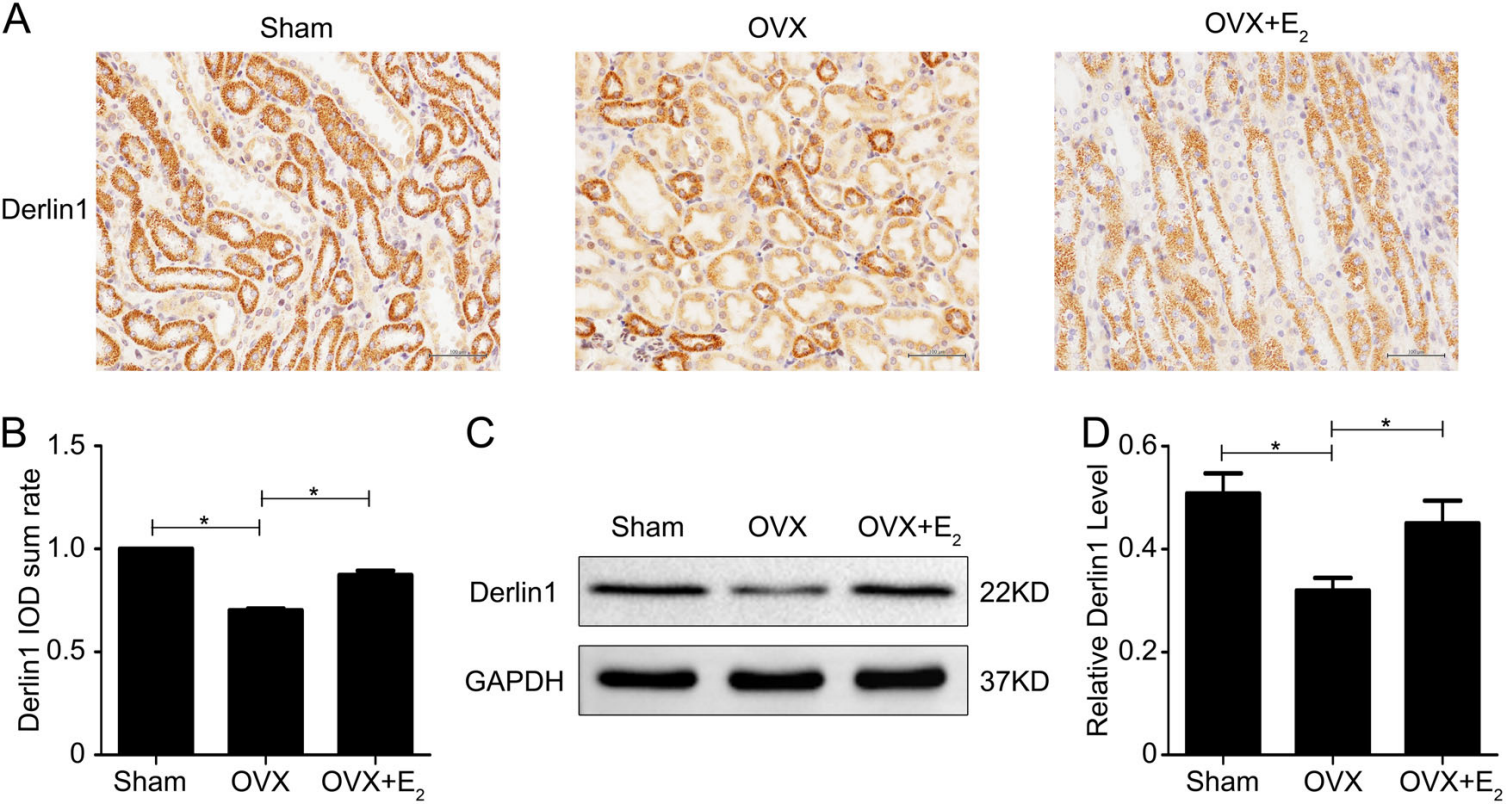
Estrogen deficiency decreases the expression of derlin-1 in the kidneys of rats. **a** The expression of derlin-1 in rat kidneys was examined by immunohistochemical staining. **b** Quantification of the positive staining for derlin-1 protein in (**a**). **c** Western blot analysis was performed to examine derlin-1 expression in kidney lysates from the different treatment groups. **d** Quantitation of the western blot data for derlin-1 expression shown in **c** normalized to GAPDH expression. The bars are the means ± SE values from three independent experiments. *P *<* 0.05

In our previous study, we showed that derlin-1, an E3 ligase mediator, promotes efficient α-ENaC ubiquitination and enhances α-ENaC ubiquitin-proteasome degradation^18^. As shown in Fig. 4a, immunochemical analysis confirmed the suppression of derlin-1 expression in OVX kidneys, but E_2_ treatment attenuated this suppression. The quantified data are shown in Fig. 4b. Western blots confirmed the reduced expression of derlin-1 protein in OVX kidney lysates and the restoration of normal derlin-1 protein levels in the kidneys of OVX+E_2_ rats compared with controls (Fig. 4c). The data from all experiments are summarized in Fig. 4d. These data indicated that E_2_ might modulate derlin-1 expression.

### Estrogen increases derlin-1 expression in mpkCCDc14 cells

**Fig. 5:**
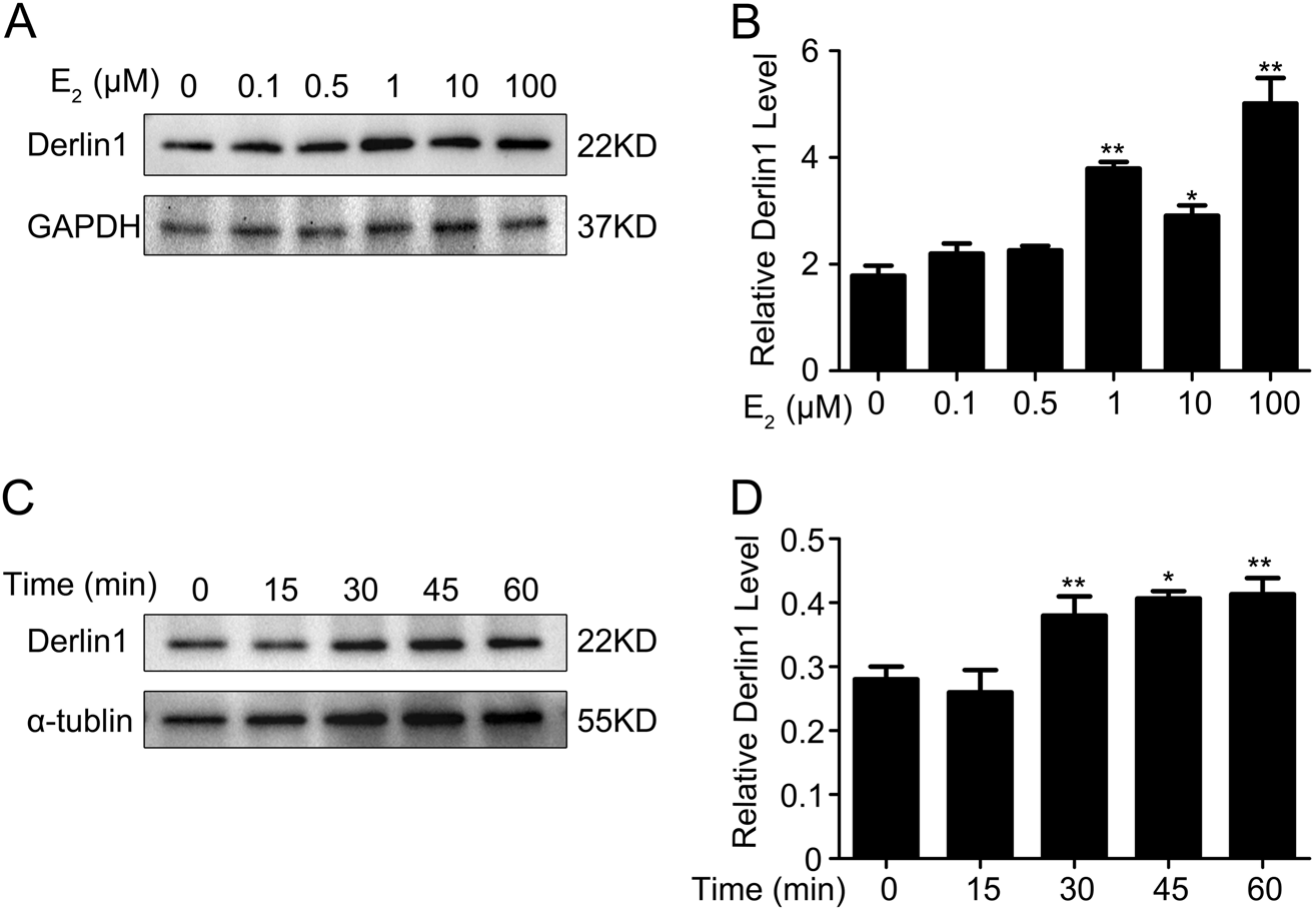
Estrogen increases the expression of derlin-1 in mpkCCDc14 cells. **a** Endogenous derlin-1 expression was dose-dependently increased by E_2_ treatment in mpkCCDc14 cells, as determined by western blot analysis. **b** Quantitation of the western blot data for derlin-1 protein shown in A normalized to GAPDH expression. The bars are the means ± SE from three independent experiments. *P *<* 0.05 and **P *<* 0.01 compared to the Con group. **c** E_2_ induced the protein expression of derlin-1 in mpkCCDc14 cells during a time course, as determined by western blot analysis. **d** Quantitation of the time-course data for derlin-1 expression shown in (**c**) normalized to α-tubulin expression. The bars are the means ± SE values from three independent experiments. *P *<* 0.05 and **P *<* 0.01 compared to the Con group

Endogenous derlin-1 protein expression in mpkCCDc14 cells was dose-dependently increased in response to E_2_ treatment (Fig. 5a). The quantified data from all experiments are shown in Fig. 5b. Western blotting also indicated a time-dependent elevation in derlin-1 expression with 100 µM E_2_ treatment in mpkCCDc14 cells (Fig. 5c). The time-course mean data for derlin-1 are shown in Fig. 5d. Derlin-1 mRNA expression was also significantly increased, both dose- and time-dependently, in mpkCCDc14 cells following E_2_ treatment (Supplementary Fig. 1c and d). These data suggested a reciprocity in α-ENaC versus derlin-1 expression in mpkCCDc14 cells in response to E_2_ treatment.

### Estrogen induces α-ENaC ubiquitination by promoting the interaction of α-ENaC with derlin-1

**Fig. 6:**
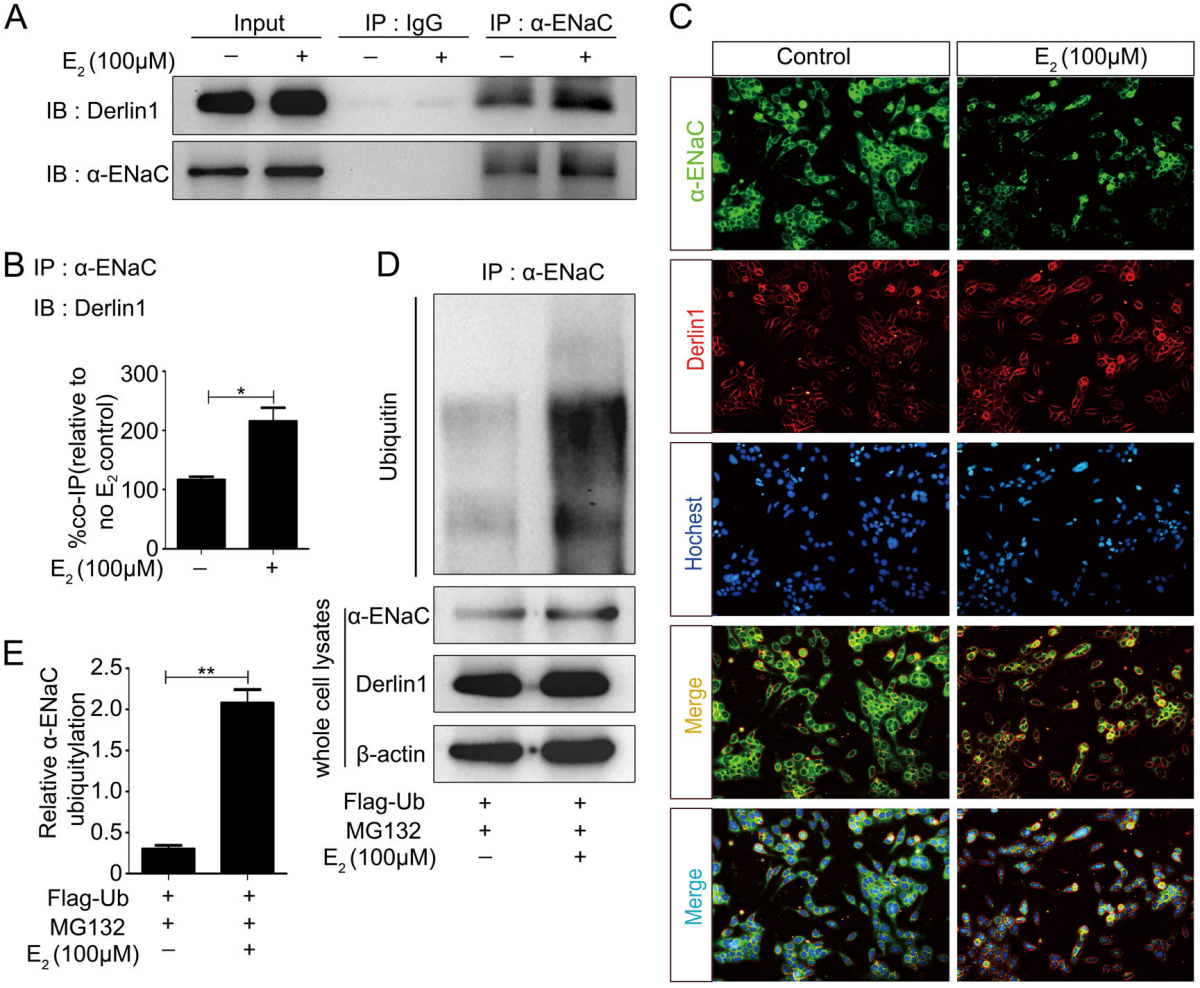
Estrogen induces α-ENaC ubiquitination by promoting the interaction of α-ENaC with derlin-1. **a** The interaction between α-ENaC and derlin-1 was detected through co-IP analysis in mpkCCDc14 cells with or without E_2_ stimulation. **b** Quantification of the binding signals from (**a**). **c** Double immunofluorescence staining was performed to confirm the interactions between α-ENaC and derlin-1 in mpkCCDc14 cells with or without E_2_ treatment. **d** More ubiquitin conjugated to α-ENaC was detected in cells treated with E_2_ than in untreated cells. **e** Quantification of the ubiquitin conjugated to α-ENaC shown in **d** normalized to β-actin expression

Our earlier study demonstrated a physical interaction between derlin-1 and α-ENaC in mpkCCDc14 cells and demonstrated a functional induction of rapid α-ENaC degradation at the posttranscriptional level^18^. Coimmunoprecipitation (co-IP) experiments with α-ENaC antibodies performed to isolate protein complexes from mpkCCDc14 cells and subsequent blotting with derlin-1 antibodies confirmed a stimulation of the interaction between α-ENaC and derlin-1 in mpkCCDc14 cells by E_2_ treatment (Fig. 6a). As summarized in Fig. 6b, E2 treatment significantly enhanced the interaction between α-ENaC and derlin-1. Double immunofluorescence staining also showed an increase in the colocalization between α-ENaC and derlin-1 in E_2_-treated mpkCCDc14 cells (Fig. 6c), in agreement with the biochemical data (Fig. 6a).

We previously demonstrated that derlin-1 promotes the rapid degradation of α-ENaC by regulating the ubiquitination of α-ENaC and that E_2_ treatment upregulates derlin-1 expression in mpkCCDc14 cells. In the present study, treatment of mpkCCDc14 cells with MG132, a proteasome inhibitor, and immunoprecipitation with α-ENaC antibodies confirmed that more than fourfold more ubiquitin was conjugated to α-ENaC in mpkCCDc14 cells treated with E_2_ than in control cells (Fig. 6d), suggesting that E_2_ promoted the ubiquitination and degradation of α-ENaC. The quantified data are shown in Fig. 6e.

**Fig. 7:**
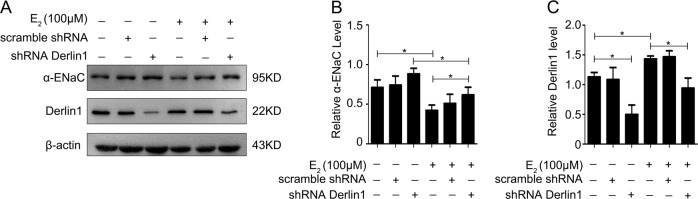
Knockdown of derlin-1 significantly reversed the E_2_-induced decrease in α-ENaC. **a** Downregulation of derlin-1 by shRNA-derlin-1 transfection in mpkCCDc14 cells significantly reversed the E_2_-induced decrease in α-ENaC. **b** Quantification of the α-ENaC expression shown in (**a**) normalized to β-actin expression. **c** Quantification of the derlin-1 expression shown in (**a**) normalized to β-actin expression. The bars are the means ± SE values from three independent experiments. *P *<* 0.05

Knockdown of derlin-1 expression in mpkCCDc14 cells by shRNA-derlin-1 transfection decreased derlin-1 expression by approximately 65% (Fig. 7a) and significantly reversed the E_2_-induced decrease in α-ENaC expression. The data regarding α-ENaC and derlin-1 expression from all experiments are summarized in Figs. 7b and c. These data indicated that E_2_ promoted ENaC degradation through a mechanism involving derlin-1.

### Estrogen induces phosphorylation of AMPK in mpkCCDc14 cells

We next investigated whether mechanisms other than estrogen-mediated ones were responsible for the suppression of α-ENaC expression in mpkCCDc14 cells.

**Fig. 8:**
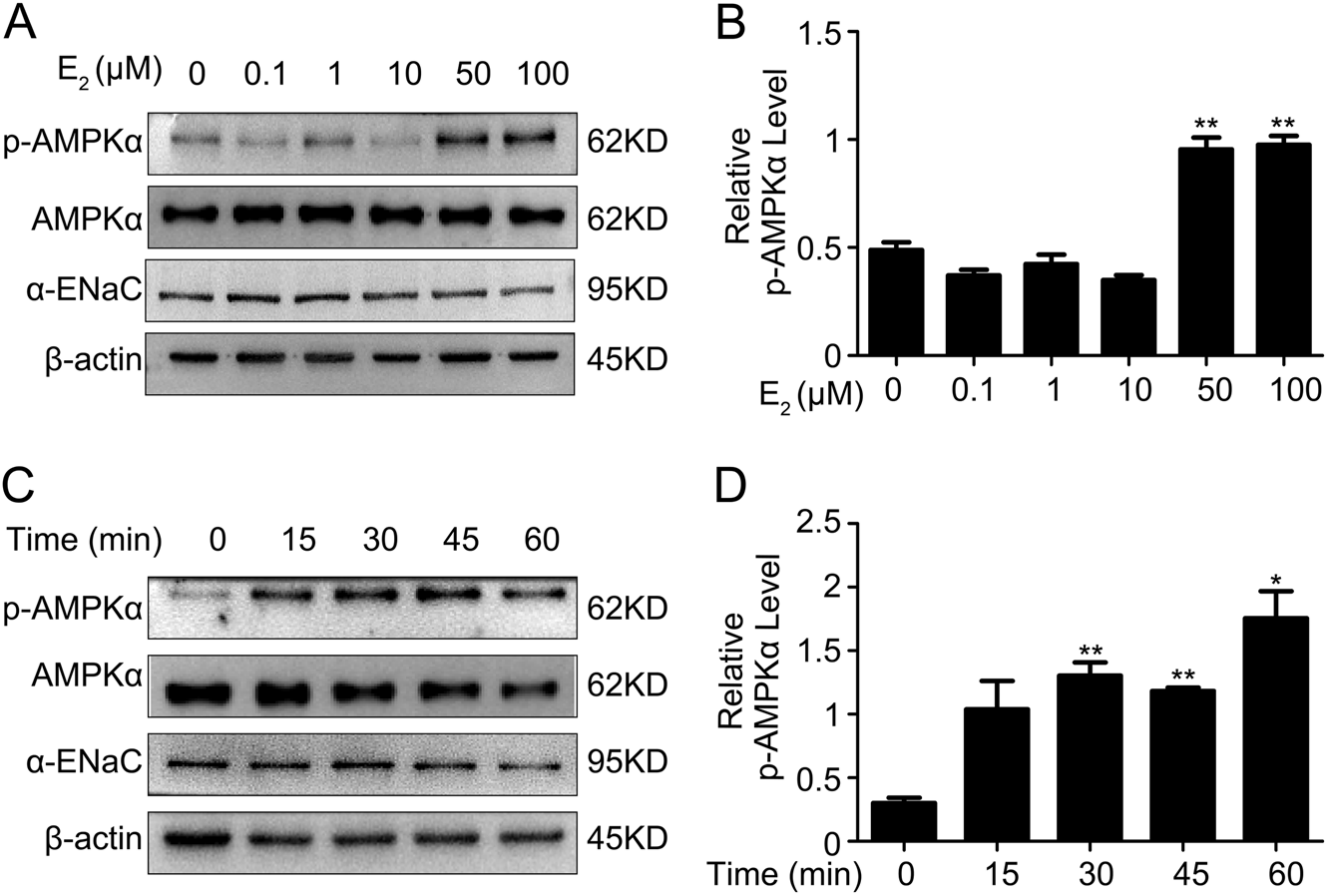
Estrogen evokes phosphorylation of AMPK in mpkCCDc14 cells. **a** The expression levels of phosphorylated AMPK and total AMPK in mpkCCDc14 cells stimulated with a series of E_2_ concentrations were determined by western blot analysis. **b** Quantification of the phosphorylated AMPK expression shown in (**a**) normalized to β-actin expression. **c** The expression levels of phosphorylated AMPK and total AMPK in mpkCCDc14 cells during a time course of E_2_ stimulation were determined by western blot analysis. **d** Quantification of the phosphorylated AMPK expression shown in **c** normalized to β-actin expression. The bars are the means ± SE values from three independent experiments. *P *<* 0.05 and **P *<* 0.01 compared to the Con group

Many studies have demonstrated that the activation of AMPK inhibits ENaC activity in both the *Xenopus* oocyte expression system and in polarized mpkCCDc14 cells^25,26^. Phosphorylation of AMPK was dose-dependently increased in mpkCCDc14 cells treated with 0.1, 1, 10, 50, and 100 μM E_2_ (Fig. 8a), and α-ENaC expression was dose-dependently decreased with these E_2_ treatments, in agreement with the results shown in Fig. 3a. The mean concentration series data on phosphorylated AMPK expression from all experiments are summarized in Fig. 8b. Similarly, E_2_ time-dependently induced AMPK phosphorylation in mpkCCDc14 cells (Fig. 8c). The mean time-course data for phosphorylated AMPK expression are shown in Fig. 8d.

### Estrogen drives the activation of AMPK-dependent inhibition of ENaC in mpkCCDc14 cells

**Fig. 9:**
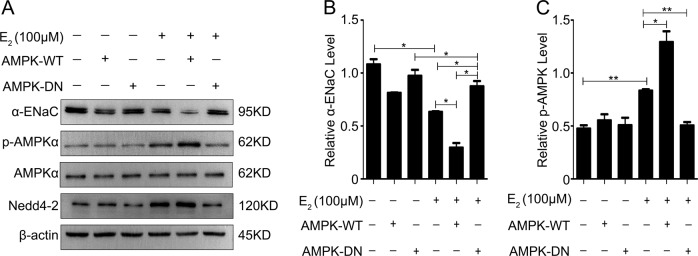
Estrogen drives activation of AMPK-dependent inhibition of ENaC in mpkCCDc14 cells. **a** The expression levels of α-ENaC, phosphorylated AMPK, total AMPK and NEDD4-2 in mpkCCDc14 cells under different conditions were determined by western blot analysis. **b** Quantification of the α-ENaC expression shown in A normalized to β-actin expression. **c** Quantification of the phosphorylated AMPK expression shown in A normalized to β-actin expression. The bars are the means ± SE values from three independent experiments. *P *<* 0.05 and **P *<* 0.01

As shown in Fig. 9a, transfection of HA-tagged WT-AMPK-α1 into mpkCCDc14 cells significantly decreased α-ENaC expression. However, this inhibition of α-ENaC expression by AMPK was blunted by transfection with AMPK-DN, a dominant-negative (DN) AMPK-α1-K45R mutant (Fig. 9a). Nedd4-2 expression showed the same responses observed for phosphorylated AMPK expression. The mean data for α-ENaC expression and AMPK phosphorylation from all experiments are summarized in Fig. 9b and c, respectively. These data indicated that the activation of AMPK by E_2_ played a role in the inhibition of α-ENaC expression.

## Discussion

Sex differences are found in a number of syndromes and diseases in addition to the differences between males and females found in many physiological functions^27–29^. For example, menopausal women have more severe manifestations of cardiovascular diseases and hypertension than men due to the physical functions of sex hormones. Many studies have indicated that sex hormones, including estrogen and progesterone, play important roles in whole-body sodium and water regulation in addition to their reproductive functions. The impacts of estrogen and progesterone exposure on systems that regulate sodium and water are particularly evident in a number of syndromes, including orthostatic hypotension, insulin resistance, polycystic ovary syndrome, and postoperative neurological conditions in women^30,31^. Receptors for estrogens are widely expressed in nonreproductive tissues, such as the hypothalamus, cardiovascular tissues, and kidney tubules, where they are involved in fluid regulation, supporting a role for estrogen in sodium and water regulation^32,33^.

Other evidence supports a role for sex hormones, such as estrogen, progesterone, and genistein, in inducing changes in uterine fluid volume and Na concentrations through regulation of the expression of α-, β-, and γ-ENaC and α-Na/K-ATPase^34^. Progesterone can upregulate the expression of ENaC and Na/K-ATPase and increase uterine fluid Na reabsorption, whereas lower expression of these proteins in response to estrogen and genistein can result in lower reabsorption of uterine fluid Na. An association between E_2_ replacement and reduced blood pressure has been reported in OVX female rats^35^. In the present animal and cell experiments, estrogen suppressed ENaC expression and function, which likely reduced reabsorption of urinary Na^+^ to lower the concentrations of serum Na^+^ and fluids and provide a balanced blood pressure.

Our findings are consistent with those of previous studies demonstrating decreased expression of Na-K-Cl cotransporter isoform 2 (NKCC2), sodium chloride cotransporter (NCC), and ENaC, along with reductions in blood pressure and aldosterone, in response to estrogen^36^. Aldosterone is a primary hormone that modulates renal tubular-regulated sodium retention under physical conditions, and water retention is followed by body sodium retention. Several studies have demonstrated that estrogen modulates the renin-angiotensin-aldosterone system (RAAS) by decreasing aldosterone and upregulating the AT_2_ receptor. Conversely, estrogen deficiency evokes RAAS activity. The evidence suggests that the protective effects of estrogen on blood pressure are related to its regulation of the RAAS pathway and aldosterone production^37,38^. In the present study, OVX rats showed higher plasma aldosterone concentrations than rats given estrogen replacement, so the elevated blood pressure in the OVX rats may have been due to the elevated aldosterone levels induced by estrogen depletion. Estrogen-related sodium retention occurs independently from potassium excretion and aldosterone regulation^4^. Our in vitro experiments support a role for estrogen in the direct regulation of sodium channels. These findings implied that estrogen affects sodium reabsorption in part through an aldosterone-independent mechanism rather than through changes in aldosterone-mediated regulation of the renal distal tubule. Other evidence for an aldosterone-independent mechanism of estrogen included the localization of estrogen receptors in the kidney tubules; therefore, the effects of estrogen could be mediated by the estrogen receptor and its downstream pathway. However, establishing the working mechanism between estrogen and aldosterone at the cellular level will require further study.

The regulation of ENaC degradation has been linked to both the lysosome-mediated and proteasome-mediated degradation pathways. Derlin-1, as an E3 ubiquitin ligase modulator of the Derlin family, recognizes target proteins of ubiquitin-mediated proteasomal degradation in the cytosol^39,40^. Our previous study showed that derlin-1 recognized α-ENaC and promoted its ubiquitin-mediated degradation in mammalian cells. Derlin-1 also physically interacted with α-ENaC and reduced its expression, while knockdown of derlin-1 expression using derlin-1 shRNA increased the expression of α-ENaC^18^. AMP-activated kinase (AMPK) has been identified as an inhibitor of ENaC because activation of AMPK decreases ENaC activity in renal epithelial cells and lung tissues^26^. Some studies have shown an enhancement of ENaC expression in the kidney, airway, and colon in AMPKα1^−/−^ mice, further supporting a role for AMPK suppression of ENaC expression^41^.

**Fig. 10:**
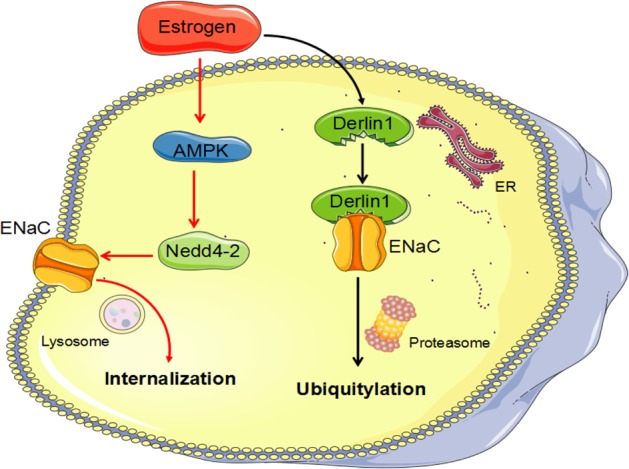
Schematic diagram showing how estrogen downregulates ENaC by promoting derlin-1 expression and AMPK activation. Estrogen treatment decreased ENaC expression through two degradation pathways: in the first, upregulation of derlin-1 by estrogen promoted ENaC proteasome-related degradation, while in the second, activation of AMPK by estrogen induced ENaC internalization from the cell membrane and increased ENaC/Nedd4-2 interaction

Inhibition of ENaC by AMPK occurs mainly due to phosphorylation by AMPK, which increases the interaction of Nedd4-2 with ENaC to promote ENaC ubiquitination and retrieval^16^. Therefore, the results from the present study suggest an involvement of estrogen in both derlin-1-mediated ubiquitination and AMPK-induced suppression of ENaC expression. As shown in Fig. 10, estrogen treatment decreased ENaC expression through two degradation pathways: in the first, upregulation of derlin-1 by estrogen promoted ENaC proteasome-related degradation, while in the second, activation of AMPK by estrogen induced ENaC internalization from the cell membrane and increased ENaC/Nedd4-2 interaction.

The significance of estrogen in the regulation of body fluid and sodium balance, as well as in hypertension, is still under investigation. Some studies have provided contrasting data indicating upregulation of ENaC expression by estrogen following acute lung injury or in the uteri of sex steroid-deficient rats^35,42^. Thus, the nature of estrogen regulation of sodium channels still requires further elucidation in terms of the physiological function of estrogen.

In our current study, we demonstrated that estrogen reduces ENaC expression and blood pressure in ovariectomized rats, and we reported that estrogen-mediated ENaC expression is decreased by derlin-1 upregulation and AMPK activation in mpkCCDc14 cells. This influence of estrogen on renal sodium channels may be associated with salt sensitivity in estradiol-depleted women, thereby providing further evidence to explain postmenopausal hypertension.

## Supplementary information

Supplementary Figure 1
