# Supplementary material for: Estrogen negatively regulates the renal epithelial sodium channel (ENaC) by promoting Derlin-1 expression and AMPK activation
Source: Exp Mol Med. 2019 May 21;51(5):55. doi: 10.1038/s12276-019-0253-z (PMC6529463; doi:10.1038/s12276-019-0253-z)

Supplemental Figure Legend

Supplementary Figure 1: Effect of Estrogen on α-ENaC and derlin-1 mRNA expressions.

The mRNA expressions of α-ENaC (A) and derlin-1 (C) was determined in mpkCCDc14 cells treated with 0.1, 0.5, 1, 10, 100 μM E_2 by real-time PCR. The mRNA expressions of α-ENaC (B) and derlin-1 (D) were also examined as a function of time during the stimulation of mpkCCDc14 cells with 100 μM E_2 by real-time PCR. Bars are means ± SE from three independent experiments, *$P<0.05$*, **$P<0.01$* compared to Con.

Supplementary Figure 1

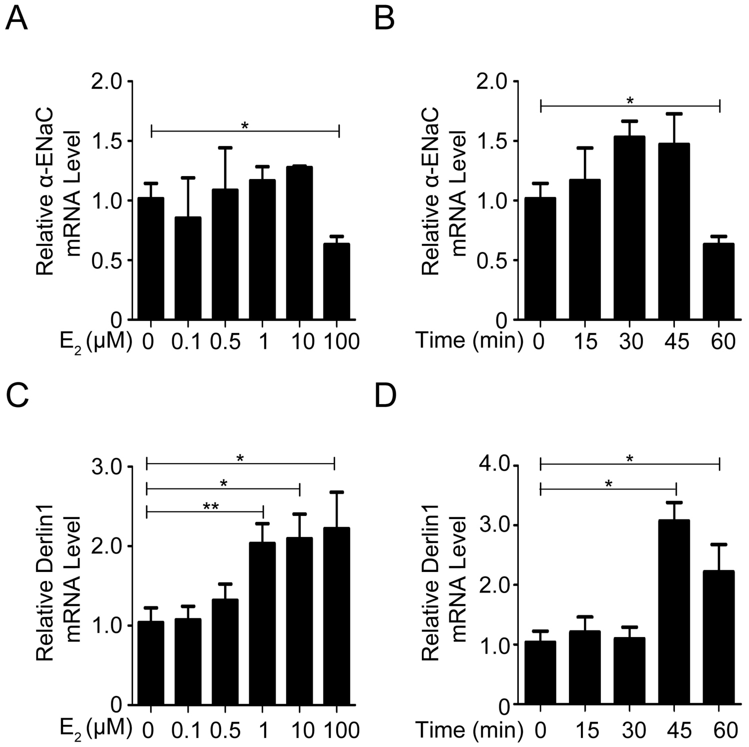

Supplement: Supplementary file 1 — Supplementary Figure 1 [file 12276_2019_253_MOESM1_ESM.pdf]
